# Autochthonous *Plasmodium vivax *malaria in a Greek schoolgirl of the Attica region

**DOI:** 10.1186/1475-2875-11-52

**Published:** 2012-02-21

**Authors:** Chariclia V Loupa, Konstantina Tzanetou, Ioannis Kotsantis, Stylianos Panopoulos, Moyssis Lelekis

**Affiliations:** 1 2nd Department of Internal Medicine, "A. Fleming" General Hospital, 14 25th March St., Melissia, Athens GR-15127, Greece; 2 Department of Microbiology, "Alexandra" General Hospital, 80 V. Sofias Ave. & 2-4 Lourou St., Athens GR-11528, Greece

**Keywords:** Malaria, Autochthonous, *Plasmodium vivax*, Greece

## Abstract

In August 2009, one case of autochthonous malaria due to *Plasmodium vivax *was diagnosed in Greece in a young woman residing in the Eastern Attica region. The source of infection could not be identified. No other autochthonous malaria cases have been described in the Attica region since 1974. This was a sporadic case with no evidence of further local transmission, and no more cases have been reported in Attica up to now, two years later.

## Background

In non-endemic countries, malaria cases are mostly imported (from travelers or immigrants), but blood transfusion malaria, or malaria in transplant recipients, or even cases of "airport malaria" can occasionally be seen [1]. Greece has been malaria free since 1974. However, rare cases of autochthonous malaria are occasionally reported. Recently, in August 2011, an announcement was posted by European Centres for Disease Prevention and Control (ECDC) and American Centers for Disease Control and Prevention (CDC) that six autochthonous malaria cases were reported in southern Greece [2,3]. An autochthonous case in a schoolgirl in the Attica region in 2009 is hereby described.

### Case presentation

**Figure 1:**
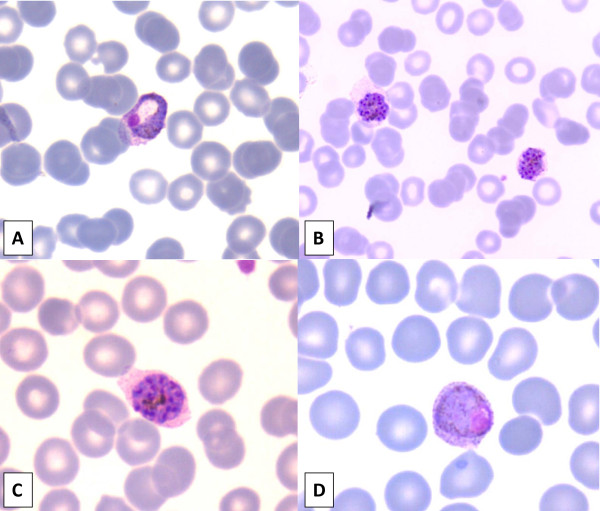
***Plasmodium vivax *in Giemsa-stained thin blood smear with all developmental stages present in peripheral blood**. **(A) **Growing amoeboid trophozoite in enlarged red blood cell (RBC) with eosinophilic stippling (schuffner's dots). **(B) **Immature schizonts with clumps of brown pigment almost fill the enlarged RBCs. **(C) **Mature schizont with merozoites (about 14) and clumped pigment. **(D) **Macrogametocyte with diffuse brown pigment and eccentric compact chromatin.

A 17-year-old young woman of Greek origin, living in the Eastern Attica region, was admitted to hospital (mid-August, 2009), because of high fever (up to 40.3°C) of abrupt onset with chills for eight days. She also reported sore throat, headache, remarkable weakness and sweating with unpleasant smell. Fever was almost periodic, being higher every second day. Anti-pyretic drugs were causing lysis with excess sweating. Her past medical history was unremarkable. She denied travelling abroad and had no history of blood transfusion. Upon admission, patient was in good condition, and physical examination revealed mild hepatosplenomegaly. There were multiple skin lesions suggesting insect bites, especially on lower limbs. During the first 48 hours of hospitalization, she had fever up to 40°C, symptomatically treated with paracetamol. Initial blood evaluation showed normocytic, normochromous anaemia (haematocrit 31.3%, MCV = 89.6 fL, MCHC = 32.3), thrombocytopaenia (platelets 48,000/μL), with a normal leukocyte count. Liver function tests yielded slightly abnormal results: AST 28 u/L, ALT 71 u/L (reference range < 40), LDH 943 u/L (reference range 230-450), and total bilirubin 1.2 mg/dL (reference range < 1,0). Kidney function tests and coagulation study were normal. ESR was 58 mm, and CRP 139 mg/L (reference range < 5). Chest x-ray and urinalysis were normal. Two sets of blood cultures and urine culture were negative. Viral (cytomegalovirus, Epstein-Barr virus), bacterial (brucella) and parasitic (*Toxoplasma, Leishmania*) serology was negative, and throat swab PCR for H1N1 antigens was also negative. Based on serology (Ra-test, ANA, anti-DNA), there was no evidence of systemic disease. Ultrasonography of the upper abdomen showed enlarged liver and spleen (17.2 and 14.1 cm, respectively). The initial Giemsa-stained peripheral blood smear was negative for *Plasmodium*. In a second peripheral blood smear, obtained on the third hospital day, *Plasmodium *parasites were identified. The patient was started on mefloquine. When the smear was re-examined by an experienced microbiologist, the parasite was identified as *Plasmodium vivax *according to morphological characteristics of trophozoites, immature/mature schizonts, and gametocytes (Figure 1A-D). Parasitaemia was approximately 3%. After completing mefloquine therapy (750 mg po ×1, then 500 mg po × 1 12 h later), the patient's condition improved and after assessment of G-6-PD levels, she was discharged on primaquine (30 mg base po ×1) for 14 days. Seen as an outpatient, she remains in good health and relapse-free to September 2011 (two years after discharge).

The young woman is of Greek origin and living in Nea Makri. This small summer holiday town of 14,809 permanent residents is located in Eastern Attica region, 32 km from the city of Athens and 12 km from "El. Venizelos" International Airport [4]. Many immigrants from endemic countries live in this area, and they work at plant nurseries located approximately 10 km from the small town. There are also still water pools and even swamps at a 10 km distance. The patient had no travel history in malaria endemic countries. She did not mention any visits to the airport, and she had no history of blood transfusion. She had a schoolmate from Africa, who was apparently healthy. A limited enquiry that was conducted did not reveal other people with malaria-like symproms in the patient's family or school.

## Discussion and conclusion

In Greece, malaria is among the mandatory reported diseases. According to official data, 20-30 new cases of malaria are reported every year [5]. Like all non-endemic countries, cases are mostly imported (from travelers or immigrants), but there are also few reports of blood transfusion malaria [1]. However, a few autochthonous cases of malaria were reported in Thrace (Northern Greek region) [6-8], in the mid- and late Nineties. Since then, there were no more autochthonous cases until 2009, when six cases of autochthonous malaria were reported (through the European Surveillance System - TESSy) and one again in 2010, all residing in the same area of Evrotas, a small agricultural and wetland area (Lakonia, Peloponnese, southern Greece) [9,10]. These cases were all caused by *P. vivax *[2]. Since June 2011, another six cases of autochthonous malaria were reported [2,3]. All six cases were diagnosed with *P. vivax *malaria. Four of these were again from Evrotas region. One case is a young Roma child, and three cases are adult Greek citizens not belonging to a minority group. The two remaining cases are in adult Greek citizens that reside close to the town of Chalkida, in the district of Evoia, where no previous autochthonous malaria cases had been reported. Both the Lakonia and Evoia districts have a large population of non-documented illegal migrant farm workers from malaria-endemic countries. In August 2011, a *P. vivax *malaria infection was diagnosed in a Romanian citizen who had been working in agriculture in Evrotas region [11].

The described autochthonous malaria case was diagnosed in August 2009. It was reported to KEELPNO (the Greek Centers for Disease Control and Prevention) through the mandatory notification system. This is the only case of autochthonous malaria in Attica (Athens city and suburbs region) before 2011. It was caused by *P.vivax*, as all the Laconia and Evoia cases, and, as previously noted, in this particular Attica area a lot of immigrants reside and work. This was a sporadic case with no evidence of further local transmission.

The most likely explanation of the few reported autochthonous cases is limited transmission of the disease to nearby residents or workers by local mosquitoes (vectors), infected from undiagnosed imported malaria cases or asymptomatic gametocyte carriers infected outside Greece. This is attributed to the large number of documented and non-documented (illegal) immigrants from endemic countries of Asia and Africa. Also, climate change, causing high temperatures during summer time, is favourable for mosquito growth [10].

Isolated autochthonous cases caused by *P. vivax, Plasmodium falciparum *and *Plasmodium ovale *have been reported in other European countries, such as in Spain, France, Italy and Germany. In Spain, a case of malaria was reported from the Aragon region in 2010 [12], and there was also one in 2001 that may have been airport malaria [13]. One case was diagnosed in Corsica, France, and two in Marseille, in spring and summer 2006 [14,15]. In Italy, there were two cases in 1997-1998, with one of them linked to an imported case from India [5,16] and one in late Eighties [17]. In Germany, there were two cases in a paediatric ward in 1997 [18]. Such cases never resulted in established local transmission involving more than a few cases.

During 2010 and 2011, attention was paid to mosquito-borne infections in Greece because of West Nile virus outbreaks and KEELPNO has responded by enhancing its surveillance system and intensifying mosquito control. Efforts to destroy mosquitoes using insecticides are currently undertaken by the Greek government [19].

Although malaria risk in Greece remains extremely low, and the country has implemented control measures, individual measures to prevent mosquito bites, such as using insect repellent when outdoors during the peak-biting period for mosquitoes (dusk and dawn), seem reasonable. Also, health practitioners must include malaria in the differential diagnosis, and try to rapidly identify and report suspected malaria cases to respective authorities. Diagnosis is based on microscopy (blood smears) and/or serology. When there is a high suspicion index for malaria, repeated peripheral blood smears should be examined, drawn every six to 12 hours for a minimum of three days, preferably upon fever or chills [20].

## Competing interests

The authors declare that they have no competing interests.

## Authors' contributions

CL and ML were attending the patient. KT was the microbiologist that identified the parasite species. IK and SP carried out the epidemiological study. All authors read and approved the final manuscript.
